# Projected Treatment Capacity Needs in Sierra Leone

**DOI:** 10.1371/currents.outbreaks.3c3477556808e44cf41d2511b21dc29f

**Published:** 2015-01-30

**Authors:** Richard A White, Emily MacDonald, Birgitte Freiesleben de Blasio, Karin Nygård, Line Vold, John-Arne Røttingen

**Affiliations:** Department of Infectious Disease Epidemiology, Norwegian Institute of Public Health, Oslo, Norway; Department of Infectious Disease Epidemiology, Norwegian Institute of Public Health, Oslo, Norway; Department of Infectious Disease Epidemiology, Norwegian Institute of Public Health, Oslo, Norway; Department of Infectious Disease Epidemiology, Norwegian Institute of Public Health, Oslo, Norway; Department of Infectious Disease Epidemiology, Norwegian Institute of Public Health, Oslo, Norway; Department of Infectious Disease Epidemiology, Norwegian Institute of Public Health, Oslo, Norway

**Keywords:** ebola

## Abstract

Background: The ongoing outbreak of Ebola Virus Disease in West Africa requires immediate and sustained input from the international community in order to curb transmission. The CDC has produced a model that indicates that to end the outbreak by pushing the reproductive number below one, 25% of the patients must be placed in an Ebola Treatment Unit (ETC) and 45% must be isolated in community settings in which risk of disease transmission is reduced and safe burials are provided. In order to provide firmer targets for the international response in Sierra Leone, we estimated the national and international personnel and treatment capacity that may be required to reach these percentages. Methods: We developed a compartmental SEIR model that was fitted to WHO data and local data allowing the reproductive number to change every 8 weeks to forecast the progression of the EVD epidemic in Sierra Leone. We used the previously estimated 2.5x correction factor estimated by the CDC to correct for underreporting. Number of personnel required to provide treatment for the predicted number of cases was estimated using UNMEER and UN OCHA requests for resources required to meet the CDC target of 70% isolation. Results: As of today (2014-12-04), we estimate that there are 810 (95% CI=646 to 973) EVD active cases in treatment, with an additional 3751 (95% CI=2778 to 4723) EVD cases unreported and untreated. To reach the CDC targets today, we need 1140 (95% CI=894 to 1387) cases in ETCs and 2052 (95% CI=1608 to 2496) at home or in a community setting with a reduced risk for disease transmission. In 28 days (2015-01-01), we will need 1309 (95% CI=804 to 1814) EVD cases in ETCs and 2356 (95% CI=1447 to 3266) EVD cases at reduced risk of transmission. If the current transmission rate is not reduced, up to 3183 personnel in total will be required in 56 days (2015-01-29) to operate ETCs according to our model. Conclusions: The current outbreak will require massive input from the international community in order to curb the transmission through traditional containment mechanisms by breaking the chains of transmission in Sierra Leone. If sufficient treatment facilities, healthcare workers and support personnel are not rapidly deployed, the increasing number of cases will be overwhelming.In addition to supporting isolation and treatment mechanisms, other viable control options, such as the development of an effective vaccine, should be supported.

## Background

Since December 2013, an outbreak of Ebola virus disease (EVD) of unprecedented size and geographic extent has been ongoing in West Africa. As of 2014-12-03, over 17,000 cases and 6,000 deaths have been reported to the World Health Organization by the three most affected countries, Guinea, Liberia and Sierra Leone^1^ . Cases have also been reported in Nigeria, Senegal, the United States, Spain and, most recently, Mali. Since its first case was reported in May 2014, WHO reports that Sierra Leone has reported 7312 cases and 1583 deaths. In order to interrupt the chains of transmission and prevent further spread, a number of control measures have been initiated, including establishing Ebola Treatment Centres (ETCs), increasing case ascertainment and contact tracing, and promoting safe burial practices^2^ . Although these approaches have been used to successfully curb previous outbreaks of EVD in Central and Eastern Africa, for the first time, EVD has spread throughout densely populated urban areas among highly mobile populations who have no previous knowledge of the disease^3^ . The current outbreak will therefore require a sustained, innovative and flexible approach to achieve containment.

The international response to this crisis has been widely criticized as slow and insufficient^4^^,^ 5 . As early as June 2014, Medecins Sans Frontieres (MSF), an organization which has been involved in providing treatment from the early stages of the outbreak, has been requesting large-scale and sustained support from the international community. Despite these requests, the response has been slow to materialize. However, on 2014-08-08, the World Health Organization declared the outbreak to be a Public Health Emergency of International Concern (PHEIC)^6^ . On 18 September the UN Security Council determined that the outbreak was a "threat to international peace and security" and announced the creation of the United Nations Mission for Ebola Emergency Response (UNMEER)^7^ . This was the first time in history that the UN has created a mission for a public health concern. Even with these actions the outbreak continued to grow in magnitude and the potential for spread to neighbouring countries is of serious concern.

Several models have been developed to forecast the progression of the outbreak, with and without intervention options. A model produced by the WHO's Ebola Response Team, published September 2014, aimed to document trends in the epidemic and project expected case numbers for the coming weeks^8^ . Based on data reported to the WHO from Guinea, Liberia, and Sierra Leone until 14 September 2014, this model concluded that there was a possibility that for the medium term EVD may become endemic among the human population of West Africa. A model produced by the CDC using data from Liberia and Sierra Leone until 29 August 2014 aimed to galvanize support for multinational intervention by demonstrating the large long-term costs of delay and giving estimates of the size of the control interventions needed^9^ . The CDC model concluded that to end the outbreak by pushing the reproductive number below one, 25% of the patients must be placed in an Ebola Treatment Centre (ETC) and 35% isolated in community settings in which risk of disease transmission is reduced and safe burials are provided^9^ . This model reinforced that the cost of delay is devastating - the number of cases doubles every 20 days, making the 70% target even harder to achieve.

Since the creation of UNMEER, the international community has committed extensive resources to controlling the outbreak, including establishing ETCs in order to ensure patients are isolated. Although only a portion of the centres are operational at this time, the ETCs are designed to provide care to suspected and confirmed cases while preventing infection of healthcare workers and members of the community. Community Care Centres have also been promoted as a means to ensure patients are isolated in areas with insufficient ETC bed capacity or in remote areas without access to ETCs. ETCs can range in size from 20 to 400 beds, while CCCs are smaller, with 8 to 15 beds per facility. UNMEER and WHO had a 60 day target of having that capacity to isolate at least 70% of patients and ensure 70% of cases who die from EVD are safely buried by 2014-12-01^1^ . These targets were based on the assumption that 25% of cases would be isolated in ETCs while 45% would be isolated in CCCs. The overall goal is to ensure isolation of 100% of cases and provide safe burials to 100% of patients who die from EVD by 2015-01-01 (90-day target). As of 2014-12-03, WHO reports that a total of 517 treatment and isolation beds are operational in Sierra Leone, of which 190 beds are provided in CCCs. In addition, there are 8 ETCs currently under construction.

While multiple efforts have been made to model and forecast the epidemic, few have explicitly quantified the number of treatment places necessary to achieve the 70% target set by the CDC. Lewnard et al. developed a transmission model to assess the effectiveness of expanding EVD treatment centres, increasing case ascertainment and allocating protective household kits in Montserrado County, Liberia^10^ . The authors found that the number of beds required to contain the outbreak substantially exceeded those pledged by the international community. However, similar projections have not been developed for Sierra Leone.

We use a flexible mathematical model to estimate the number of treatment places, personnel and equipment needed to obtain the 70% target set by the CDC in Sierra Leone over the next two months (from 2014-12-04 to 2015-01-29) in order to provide firmer targets for the international response. We also reexamine what targets for treatment or isolation within a specified time frame are necessary to acheive a reproductive number below one.

## Methods

Outbreak data

To fit our model, we used the public data released by the World Health Organization^1^ from 2014-03-22 until 2014-08-13, after which we obtained approximately daily counts from the public press releases from the Sierra Leonean Ministry of Health (2014-05-27 to 2014-12-03)^11^ . For the purposes of our models, we considered all confirmed, suspected, or probable cases to be EVD cases.

Model

We developed a compartmental model to describe the outbreaks in Sierra Leone (Figure 1). Briefly, the population is divided into six compartments, with average rates and average time periods taken from the recently published WHO Ebola Response Team model^8^ .

Susceptible individuals (S) may become Exposed (E) after contact with infectious material. After an average of 11.4 days (\begin{equation*}\tau_a\end{equation*}), Exposed persons (E) then transition into non-reported Infected persons (I). Infected persons (I) may become Treated (T) after an average of 5 days (\begin{equation*}\tau_{I \rightarrow T}\end{equation*}) (in which case they are registered as an EVD case and become non-infectious), or they may Recover (R) after an average of 5+11.8 days (\begin{equation*}\tau_{I \rightarrow R}\end{equation*}), or Die (D) after an average of 5+4.2 days (\begin{equation*}\tau_{I \rightarrow D}\end{equation*}). Treated (T) persons may either Recover (TR) after an average of 11.8 days (\begin{equation*}\tau_{T \rightarrow TR}\end{equation*}) or Die (TD) after an average of 4.2 days (\begin{equation*}\tau_{T \rightarrow TD}\end{equation*}). The case fatality rate was taken to be 70%^8^ . The recently published CDC model estimated that the reporting quotient was set to 1/2.5=40% (on 2014-08-25)^9^ . We used this number to crudely assume that 40% of the population have been reported, and then treated in some fashion (either submission to an ETC or isolated in a community setting or at home). Therefore, we estimated probability of Infected persons (I) becoming Treated (T) (\begin{equation*}P_T\end{equation*}) to be 40%. Due to poor data quality, we could not assign a certain proportion to ETCs versus community isolation versus home isolation and thus considered that all cases were non-infectious after being reported.

**SEIR model:**
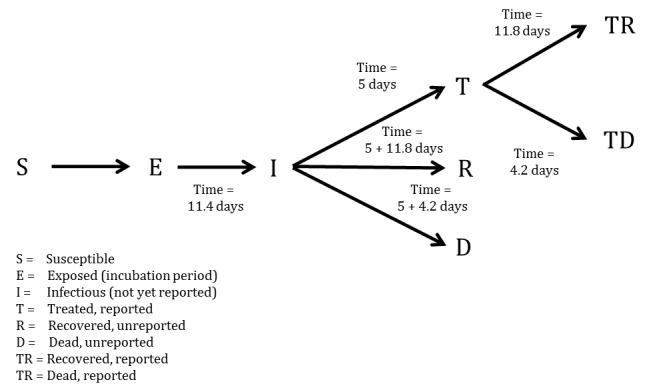

In mathematical terms, the transition equations describing the model are given as:

\begin{equation*}\frac{dS}{dt}=-\frac{\beta I S}{N}\%0A\end{equation*}

\begin{equation*}\frac{dE}{dt}=\frac{\beta I S}{N} - aE\end{equation*}

\begin{equation*}\frac{dI}{dt}=aE - \gamma_{I \rightarrow}I\end{equation*}

\begin{equation*}\frac{dR}{dt}=\gamma_{I \rightarrow R}I\%0A\end{equation*}

\begin{equation*}\frac{dD}{dt}=\gamma_{I \rightarrow D}I\end{equation*}

\begin{equation*}\frac{dT}{dt}=\gamma_{I \rightarrow T}I - (\gamma_{T \rightarrow TR} + \gamma_{T \rightarrow TD})T\end{equation*}

\begin{equation*}\frac{dTR}{dt}=\gamma_{T \rightarrow TR}T\end{equation*}

\begin{equation*}\frac{dTD}{dt}=\gamma_{T \rightarrow TD}T\end{equation*}

Where

\begin{equation*}a = \frac{1}{\tau_a}\end{equation*}

\begin{equation*}\tau_{I \rightarrow} = (1-P_T) \times 0.3 \times \tau_{I \rightarrow R} + (1-P_T) \times 0.7 \times \tau_{I \rightarrow D} + P_T \tau_{I \rightarrow T}\end{equation*}

\begin{equation*}\gamma_{I \rightarrow} = \frac{1}{\tau_{I \rightarrow}}\end{equation*}

\begin{equation*}\gamma_{I \rightarrow R} = \gamma_{I \rightarrow} \frac{(1-P_T)\times 0.3 \times \tau_{I \rightarrow R}}{\tau_{I \rightarrow}}\end{equation*}

\begin{equation*}\gamma_{I \rightarrow D} = \gamma_{I \rightarrow} \frac{(1-P_T)\times 0.7 \times \tau_{I \rightarrow D}}{\tau_{I \rightarrow}}\end{equation*}

\begin{equation*}\gamma_{I \rightarrow T} = \gamma_{I \rightarrow} \frac{P_T\tau_{I \rightarrow T}}{\tau_{I \rightarrow}}\%0A\end{equation*}

\begin{equation*}\tau_{T \rightarrow} = 0.3 \tau_{T \rightarrow TR} + 0.7 \tau_{T \rightarrow TD}\end{equation*}

\begin{equation*}\gamma_{T \rightarrow} = \frac{1}{\tau_{T \rightarrow}}\end{equation*}

\begin{equation*}\gamma_{T \rightarrow TR} = \gamma_{T \rightarrow} \frac{0.3 \tau_{T \rightarrow TR}}{\tau_{T \rightarrow}}\%0A\end{equation*}

\begin{equation*}\gamma_{T \rightarrow TD} = \gamma_{T \rightarrow} \frac{0.7 \tau_{T \rightarrow TD}}{\tau_{T \rightarrow}}\%0A\end{equation*}

\begin{equation*}\beta = R0 \gamma_{I \rightarrow}\end{equation*}

More concisely, the above average times, rates, and probabilities are clearly defined in Tables 1-3, with sources listed.

**Figure:**
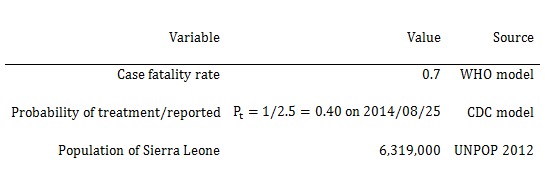
**Table 1. Probabilities, proportions, and populations in the SEIR model**

**Figure:**
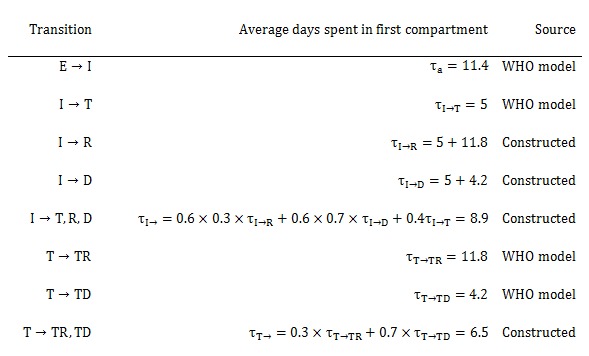
**Table 2. Average days spent in first compartment of the SEIR model when transitioning**

**Figure:**
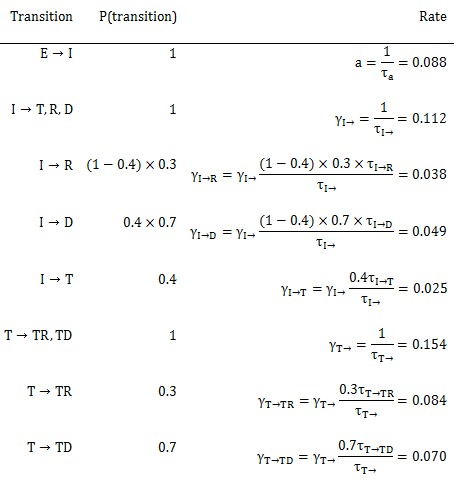
**Table 3. Rates used in the SEIR model**

Model Fitting

To estimate Reff and beginning starting values for the number of persons in compartments E and I, we implemented an ensemble trajectory model with the effective reproductive number allowed to change every 2*28=56 days (all other parameters were fixed and based from either the WHO or CDC model). The choice of 56 days was made based on preliminary simulations. When investigating the effect of varying the time interval with fixed transmissibility, we found that allowing the parameters to change every 28 days was too flexible and gave poor forward projections, while 72 days was not flexible enough and gave a poor model fit. Briefly, a matrix of plausible parameter values were generated (Reff=0.7, 0.8, ..., 2.4; E=2, 12, ..., 82; I=2, 12, ..., 82). For each parameter combination (Γ), the above model was fitted for the first 56 days (i.e. after fixing all other parameters from the WHO and CDC models, we estimated the effective reproductive number in 56 day windows). We then evaluated the fit of the model using the following formula (\begin{equation*}lddp\end{equation*} is the least deviation per datum):

$$ldpp(\Gamma)=\frac{1}{N} \sum_{i=1}^N |Obs_i-Est_i |$$

Where $Obs_i$ was the observed cumulative number of cases at data point i, and $Est_i$ was the estimated cumulative number of cases at data point i.

From this, we calculated the probability that the outbreak was caused by each parameter combination:

$$P(outbreak was caused by \Gamma) =  \frac{1}{C}exp \left(- \frac{lddp(\Gamma)-min(lddp(\Gamma)}{2 \sigma^2} \right)$$

Where C was a normalisation constant, and \begin{equation*}\sigma=sqrt(0.2 \times (lddp(\Gamma)-min(lddp(\Gamma)))\end{equation*}. The theory behind this model is detailed elsewhere^12^ .

Briefly, this model is a form of approximate Bayesian computation, which is essentially a Bayesian model with a non-informative prior that exchanges mathematical elegance for a high degree of computational work. To describe this model in a typical Bayesian form, we would explain that we fit a non-informative prior on the effective reproduction number, allowing it to vary between 0.7 and 2.4, changing every 56 days. We would then construct a likelihood for the model and attempt to estimate a plausible distribution for Reff. As we allowed the effective reproductive number to vary every 56 days, it was difficult to construct a likelihood for the model and even more difficult to obtain convergence. Approximate Bayesian computation is useful in such situations, as one simply runs the model for all plausible values of the parameters (in this case, Reff), and retains parameter combinations that result in model estimates within a prespecified band of tolerance surrounding the outcome variable. These saved parameter combinations form the posterior distribution of the estimated parameters. Bettencourt's form of approximate Bayesian computation is a less computationally intensive form of approximate Bayesian computation, which allows for a gradiated band of tolerance instead of a clear accept/reject criterion.

For each parameter combination that had support in the first 56 days of the outbreak, we fitted another 18 models (Reff=0.7, 0.8, ..., 2.4) and repeated the same procedure. This algorithm was run until it reached the end of the reported data, at which point the probability of the outbreak being caused by each trajectory was calculated. Each trajectory was then forecast to the present day (2014-12-04) and 56 days beyond, with estimated probabilities assigned.

To obtain estimates for each compartment, the differential equations listed above were solved using the "lsoda" function in R (version 3.1.1). From the compartmental model, we extracted the number of estimated cases, new estimated cases each day, estimated reported cases, new estimated reported cases each day, exposed persons currently in the incubation period, EVD cases currently in treatment and non-infectious, and EVD cases currently unreported and infectious in the community.

Intervention

We implemented a simpler version of the CDC's recommended proportions (25% to ETCs and 45% in community centres) of isolated cases to test their efficacy. Again due to poor data quality and uncertainty regarding the situation on the ground, we considered that all cases were grouped together in a single non-infectious state after being reported. We investigated the impact of 0, 10, ..., 90, 100% of infectious EVD cases receiving treatment or being isolated after 0, 1, ..., 10 days on average. This is in contrast to the baseline projection where 40% are treated (i.e. considered reported and non-infectious) after an average of 5 days. We observed which of the interventions resulted in a reproductive number below one, which is necessary for ending the epidemic.

Quantification of resources needed

Most organizations calculate that upwards of 110 healthcare personnel, including doctors, nurses and nurses' aids, and 100 other personnel, including logistics, water and sanitation, waste teams, cooks, laundry and cleaners, drivers and security are required for a 100-bed ETC^13^ . For the purposes of this model, four types of personnel are included: doctors, nurses/paramedics, hygienists and support staff.

## Results

Model fit

From a visual observation, the model fit is sufficient (Figure 2). We also performed a goodness of fit test by regressing the new case residuals (new daily predicted cases minus new daily observed cases) against time, and found there to be no significant slope nor intercept.

**Total estimates of cumulative cases (per 2014-12-04):**
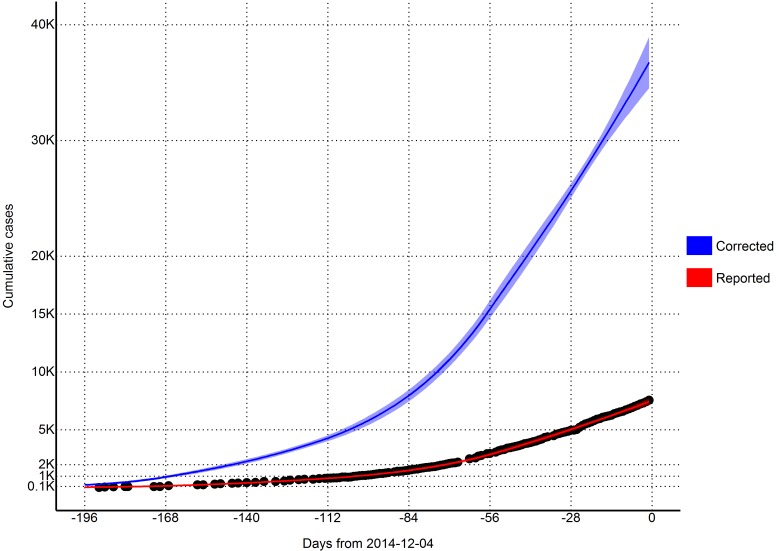

Reproductive number

Using the last 56 days of outbreak data (2014-10-09 to 2014-12-03), we estimated that the effective reproductive number was 1.1 (95% CI=0.95 to 1.24) in Sierra Leone. Reporting for the entire outbreak, the reproductive number was estimated to be 2.13 from 2014-05-22 to 2014-06-18, 1.32 from 2014-06-19 to 2014-08-13, 1.58 from 2014-08-14 to 2014-10-08, and 1.1 from 2014-10-09 to 2014-12-03.

**Table 4. Model estimates:** 

Variable	2014-12-04	2015-01-01	2015-01-29
Days from today	0	28	56
Cases (reported)	7528	10378	13665
	(7206, 7850)	(9053, 11703)	(10472, 16857)
New daily cases (reported)	95	109	126
	(70, 119)	(59, 159)	(41, 211)
Cases (corrected for underreporting)	37191	50415	65701
	(34838, 39543)	(42551, 58278)	(48134, 83267)
New daily cases (corrected for underreporting)	439	505	586
	(301, 577)	(242, 769)	(150, 1023)
Incubation period	5017	5776	6702
	(3418, 6615)	(2743, 8809)	(1682, 11722)
Infectious and unreported	3751	4308	4987
	(2778, 4723)	(2321, 6295)	(1597, 8378)
Undergoing treatment	810	928	1072
	(646, 973)	(558, 1298)	(416, 1728)
Beds for CDC target of 25% cases in ETC	1140	1309	1515
	(894, 1387)	(804, 1814)	(651, 2378)
Beds for CDC target of 45% cases in reduced transmission	2052	2356	2727
	(1608, 2496)	(1447, 3266)	(1173, 4281)
Reporting (treatment) quotient	40%	40%	40%

Predictions

Per today (2014-12-04), we estimate that there are 810 (95% CI=646 to 973) EVD active cases in treatment, with an additional 3751 (95% CI=2778 to 4723) EVD cases unreported and untreated (Table 4). Furthermore, we have estimated that there are 439 new symptomatic cases every day (Table 4).

If the outbreak continues with a reproductive number of 1.1 (95% CI=0.95 to 1.24), in 28 days (2015-01-01) this number will increase to 505 new cases every day, corresponding to a total of 50415 (95% CI=42551 to 58278) cumulative total cases (Table 4). In a further 28 days (2015-01-29) this will increase to 586 new cases every day, corresponding to a total of 65701 (95% CI=48134 to 83267) cumulative total cases (Table 4).

Achieving CDC targets for 70% containment

To reach the CDC targets today, we need 1140 (95% CI=894 to 1387) cases in ETCs and 2052 (95% CI=1608 to 2496) at home or in a community setting such that there is a reduced risk for disease transmission through effective isolation (Table 4 and Figure 3). If the outbreak continues with a reproductive number of 1.1 (95% CI=0.95 to 1.24), in 28 days (2015-01-01), we will need 1309 (95% CI=804 to 1814) EVD cases in ETCs and 2356 (95% CI=1447 to 3266) EVD cases at reduced risk of transmission (Table 4 and Figure 3). In a further 28 days (2015-01-29) we will need 1515 (95% CI=651 to 2378) EVD cases in ETCs and 2727 (95% CI=1173 to 4281) cases at reduced risk of transmission (Table 4 and Figure 3).

**Estimates of needed capacity corresponding to the CDC goals of 25% of cases in ETCs and 45% of cases in reduced transmission settings (per 2014-12-04):**
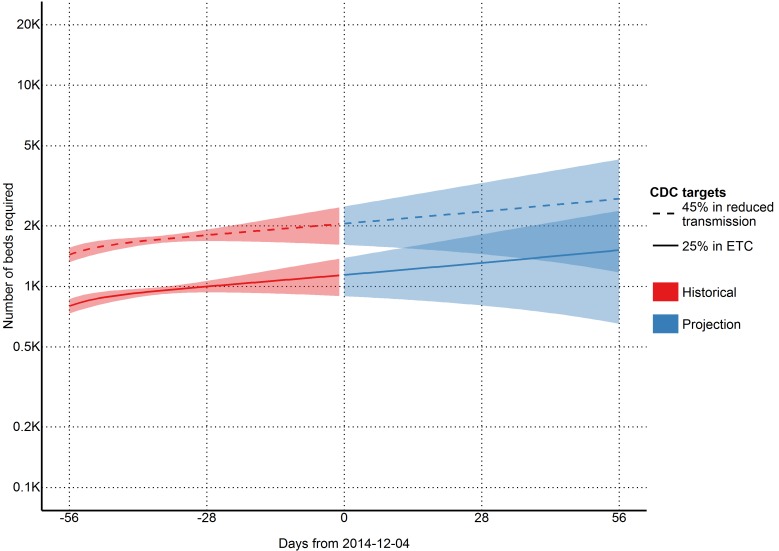

Quantification of resources needed

As of today (2014-12-04), we estimate that 2394 staff members are needed to operate ETCs that cover 25% of cases in Sierra Leone (Figure 4). This includes 114 doctors, 570 hygienists, 570 nurses/paramedics, and 1140 support staff. If this incidence of cases increases as projected and the targets of 25% of cases in ETCs is maintained, 2748 staff members will be required in 28 days (2015-01-01), and 3183 staff members will be required in an additional 28 days (2015-01-29).

**Estimates of personnel needed to operate ETCs with CDC goal of 25% of cases in ETCs (per 2014-12-04):**
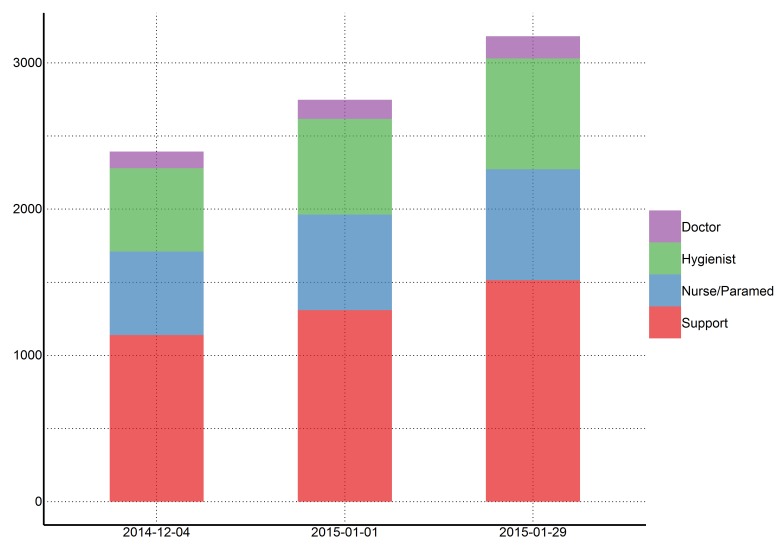

Intervention

We found that 70% containment/treatment after the five days baseline was sufficient to contain the epidemic. More specifically, 40% containment/treatment after one day, 50% after three days, 60% after five days, 70% after six days, and 90% after seven days were all sufficient scenarios (Figure 5). However, these numbers are based upon the uncertain estimate that currently 40% of cases are non-infectious, which may not be applicable.

**Ability of different intervention scenarios to achieve a reproductive number below one (per 2014-12-04):**
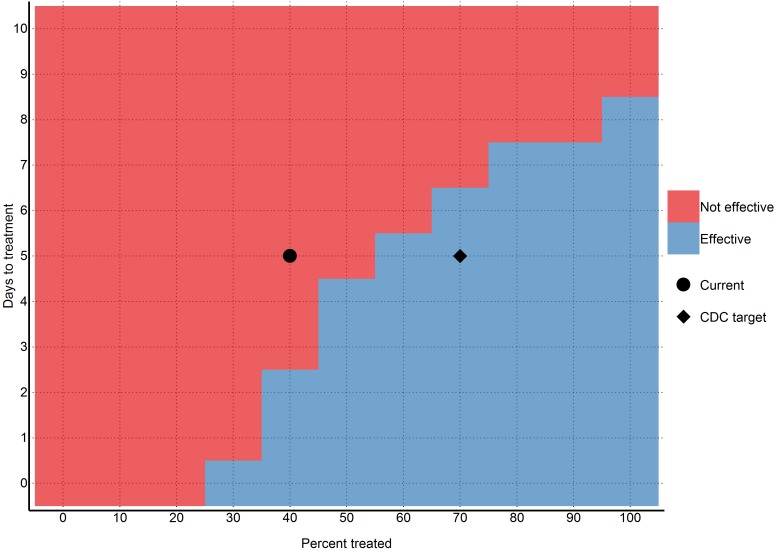

Detailed results and projections

More detailed results (including daily projections for the next six months) can be found in the appendix.

## Discussion

As of 2014-12-04, the outbreak of EVD in Sierra Leone is not yet under control and the incidence continues to increase. Although there are significant challenges associated with collecting and interpreting data in this context, our findings reinforce that the capacity for treatment and isolation is not yet sufficient to successfully break the chains of transmission in Sierra Leone.

Although the most recent Ebola Response Roadmap Situation Update suggests that more than 70% of cases are being isolated in most affected districts, only 35% of planned ETC bed capacity and 16% of planned CCC bed capacity are operational in Sierra Leone^1^ , Our model indicates that there are currently 810 (95% CI=646 to 973) cases in treatment, with an additional 3751 (95% CI=2778 to 4723) cases unreported, which surpasses the 707 beds that are currently available, of which 517 are in ETCs and 190 are in CCCs. Our model indicates that if 1140 (95% CI=894 to 1387) cases in ETCs and 2052 (95% CI=1608 to 2496) cases in reduced transmission is not achieved, the reproductive number will not be pushed below one. If the planned treatment and isolation facilities do not rapidly become operational, the situation will be further exacerbated, with the number of patients requiring isolation facilities reaching 1309 (95% CI=804 to 1814) cases in ETCs and 2356 (95% CI=1447 to 3266) cases in CCCs in 56 days.

As the available data indicates considerable variability in incidence sub-nationally, more flexible approaches to isolation and treatment may be required. There are indications that ETCs in some areas are not operating at full capacity as control measures have been successful in some districts. As the establishment of ETCs can be time-consuming and the facilities themselves can be difficult to relocate to meet needs of the developing outbreak, delays associated with establishing semi-permanent structures as ETCs may ultimately lead to further spread of the outbreak. It is also imperative to ensure that resources are distributed in areas with most need, which is an inherent challenge as the epidemiology of the outbreak changes. Our model reinforces that delays in intervention will lead to substantial increases in the required treatment capacity and manpower and that the speed of isolation after symptom onset among cases reduces the proportion that need to be isolated in order to reduce transmission. Other models have also highlighted the importance of ensuring sufficient resources to provide isolation for infected individuals within 4 days of symptom onset^14^ .

Organizations such as MSF are now reporting that new approaches that allow for rapid and flexible response are required at this stage in the outbreak, allowing response teams to be rapidly deployed to hotspots^15^ . UNMEER will be developing a district-by-district approach to ensure that sufficient isolation capacity is available in all areas of the affected countries^16^ . Our findings also suggest that if transmission is not reduced, the number of international staff required to run ETCs will increase substantially and it is likely that it will be difficult to recruit sufficient international staff to run ETCs that can accommodate 25% of patients. As the lack of CCC bed capacity is particularly notable in the WHO status updates, more focus on community-based interventions may address some of the shortfalls of ETCs. Although CCCs often have less bed capacity than ETCs, they are frequently placed in areas not served by ETCs and can alleviate pressure in areas where ETCs are at capacity^17^ . In addition, fewer personnel are required to run CCCs than ETCs as in CCCs basic patient needs such as food are generally provided for by friends and family of patients rather than by CCC staff. Although ETCs are an essential component of the response, increased coverage by CCCs will potentially allow for a more rapid response and better access to isolation facilities in remote areas.

One significant challenge with outbreak models is that they become outdated very quickly as the outbreak progresses. We therefore present today's numbers (1140 (95% CI=894 to 1387) cases in ETCs and 2052 (95% CI=1608 to 2496) cases in reduced transmission) as the minimum needed to reverse the outbreak. This is because even if the outbreak slows, today's numbers will still need to be achieved to push the reproductive number below one, unless another way of reducing transmission is found. Thus in the absence of effective vaccines or other preventative measures, 1140 (95% CI=894 to 1387) cases in ETCs and 2052 (95% CI=1608 to 2496) cases in settings leading to reduced transmission should remain as firm minimum targets for the international community, and will likely need to be exceeded given the growing context of the outbreak.

Our model allows for 56 day changes in the effective reproductive number, however, this is not analysed in a manner that allows a parametric trend to predict into the future. Instead, the effective reproductive number from the last 56 day period is extrapolated out into the future. We chose this approach for two reasons: firstly, from analysing piecewise 56 day periods, we found that the effective reproductive number varied in such a way that was not amenable to a parametric model. Secondly, with the outbreak response changing so rapidly, we did not think that using trend data from 100 days ago would be applicable to current data.

This study has a number of limitations. First and foremost, we model at the country level and only include data from Sierra Leone. This masks many geographical variations that may be happening at a more discrete level. In addition, it is highly likely that our lack of contact heterogeneity will result in overestimations of the outbreak. For this reason, we urge the reader to primarily focus on the results for "today," which are not susceptible to future overestimations. Secondly, we assume that registered cases are in treatment and thus non-infectious. While it was assumed by the WHO model that hospitalised cases were non-infectious, it is well documented that healthcare workers are continually being infected (although it has been noted that the majority of the healthcare workers were infected at home or in their local community). We are also uncertain as to our assumption that registered cases are in treatment; considering the overwhelmed nature of the West African health system. It is entirely likely that a great number of the new cases come from counting dead bodies. Furthermore, we do not account for the temporal changes in available treatment capacity in Sierra Leone. However, we believe that in the context of the goals of this study, these assumptions are acceptable. As our goal was to convert the CDC's soft target (in percentages) to a hard target of number of cases needing treatment, any temporal changes or misspecification of the model should be accounted for in our estimation of the effective reproductive number. For example, if there is a dramatic increase in treatment capacity, our estimated effective reproductive number should drop. Our assumption that reported individuals are not infectious will cause the effective reproduction number to rise. However, as long as the estimated number of cases concurs with the reported numbers, our model has met its goal in estimating the number of people currently alive with EVD. It is because of this that we choose not to focus our discussion on the effective reproductive number, nor compare with other models. In addition, this model is based on reported case data, which has significant underreporting that varies over time and geographical region. Our model attempts to correct for underreporting, however, it is not possible to validate how accurately we have done so. Finally, our model assumes that the outbreak will continue growing as it has in the past - while unlikely, the recent international efforts may have had some effect that will take place in the near future.

One issue with our model is that it was fitted using cumulative case counts, which contains autocorrelation in the errors and will yield biased estimates. Considering the goals of the model, we believed that this was the best choice. As our goal was to obtain estimates surrounding the current condition, our fitting procedure ensured that the most weight was given to the most recent (and largest) observations. However, our future predictions are most likely biased and should be treated with scepticism.

Widely reported changes have occurred in a number of areas of this epidemic: social distancing, treatment, burials, etc. We have limited information on these changes, and instead decided to base our model on two high quality sources of data (the WHO and CDC models) in favour of vague descriptive statistics that change frequently. By allowing our effective reproductive number to change every 56 days, we hope that our model will capture some of these changing parameters, which can be seen by the high quality fit of our model to the observed data. Our model assumes that after five days, 40% of the cases will be non-infectious due to some sort of treatment. If this assumption is incorrect, then our reproductive number estimate and Figure 5 will also be incorrect. However, our estimates for the number of cases (and thus the number of beds needed to reach the CDC targets) will still be correct, as this assumption only affects the relationship between the number of currently infectious cases and how they infect the susceptible. It does not affect the overall number of cases currently alive. These unaffected numbers are then multiplied against the 25%/45% CDC numbers and we obtain our estimates, independent of whether our treatment proportion or reproductive number are correctly estimated. If the CDC 2.5x (40%) underreporting figures are incorrect (which is highly likely, given that it was estimated at one point, many months ago), then the estimated numbers of people in "treatment" (i.e. uncorrected for underreporting) can then be interpreted as the number of EVD cases still alive and needing treatment. These numbers should be approximately correct, given the high quality fit of the model to the observed data.

## Conclusion

The largest outbreak of EVD in history is ongoing in Sierra Leone, with no indications as of 2014-12-04 that incidence is decreasing. Although concerted effort is being made by local authorities and the international community to stop transmission, current treatment and isolation facilities are insufficient. If the outbreak continues to spread, the response will be further complicated as the number of cases will overwhelm planned interventions. ETCs may not be flexible enough to accommodate the changing epidemiology of the outbreak and the number of international staff required to run sufficient facilities will be difficult to recruit. If the urgent need for isolation and treatment is not met, other control measures must be available, including community-based approaches to treatment and isolation, as well as development of a vaccine that can protect the susceptible population.

## Appendix 1

### Model estimates of epidemic progression

Data available on figshare: http://dx.doi.org/10.6084/m9.figshare.1298095

### Model estimates of staff requirements

Data available on figshare: http://dx.doi.org/10.6084/m9.figshare.1298096
